# Ankle Taping Does Not Affect Running Kinematics During a Treadmill Protocol in Well-Trained Runners: A Secondary Analysis from a Randomized Cross-Over Controlled Trial

**DOI:** 10.3390/jcm13226740

**Published:** 2024-11-08

**Authors:** Federico Salniccia, Javier López-Ruiz, Guillermo García-Pérez-de-Sevilla, Ángel González-de-la-Flor, María García-Arrabé

**Affiliations:** Department of Physiotherapy, Faculty of Sports Sciences, Universidad Europea de Madrid, 28670 Madrid, Spain; federico.salniccia@universidadeuropea.es (F.S.); guillermo.garcia@universidadeuropea.es (G.G.-P.-d.-S.); angel.gonzalez@universidadeuropea.es (Á.G.-d.-l.-F.); maria.gararrabe@universidadeuropea.es (M.G.-A.)

**Keywords:** ankle taping, running kinematics, rigid tape, kinesiotape, treadmill, biomechanical analysis

## Abstract

**Background:** The purpose of this randomized cross-over controlled trial was to evaluate the biomechanical effects of ankle taping using rigid tape (RT) or kinesiotape (KT) compared to no taping during treadmill running in well-trained amateur runners. **Methods**: A total of 22 participants (15 men and 7 women) completed three running sessions on a treadmill, each lasting 30 min, under different conditions: no taping (CG), RT, and KT. Sagittal and frontal plane kinematics were analyzed using the Kinovea software to assess the ankle dorsiflexion, knee flexion, hip extension, tibial angle, foot strike pattern, heel eversion, and pelvic drop across three intervals (0–10, 10–20, and 20–30 min). **Results**: The results demonstrated no significant differences in sagittal plane variables (ankle dorsiflexion, knee flexion, hip extension, and cadence) or frontal plane variables (heel eversion and pelvic drop) between the CG, RT and KT groups at any time point. Although heel eversion significantly increased over time due to fatigue, the taping conditions did not affect running kinematics. **Conclusions**: These findings suggest that neither RT nor KT alters running biomechanics in well-trained runners over prolonged treadmill running. The study highlights that taping, commonly used to prevent ankle injuries, does not significantly modify lower limb kinematics in the absence of injury. Further research is needed to evaluate the effects of taping in novice or injured runners and under more demanding conditions, such as overground running.

## 1. Introduction

Running is one of the most widely practiced physical activities worldwide, with an estimated 20% to 25% of the active population engaging in running-related activities [1]. This popularity is partly due to its accessibility, as it can be performed almost anywhere and by people of all ages and fitness levels (1). Its low cost and ease of integration into daily routines further contribute to its growing appeal, both recreationally and competitively [1].

However, this high participation rate is associated with a significant prevalence of injuries, particularly in the lower limbs. The incidence of lower limb injuries among runners varies considerably, with studies reporting that between 19.4% and 79.3% of runners experience at least one injury per year, with the ankle being one of the most frequently affected joints [2]. Globally, the ankle is the second most frequently injured body region in sports, following the knee, with ankle sprains being the most common type of injury [3].

Ankle sprains are, in fact, one of the most common sports injuries, with the highest recurrence rate among lower limb injuries. The incidence of sports-related ankle injuries ranges between 25% and 50% [4]. Furthermore, it has been observed that approximately 50% of patients with acute ankle sprains do not seek medical attention, leading to recurrences and, in many cases, chronic ankle instability (CAI). If not properly managed, these injuries can increase the risk of relapse and the development of chronic instability [5].

In this context, therapeutic interventions aimed at improving ankle stability have garnered considerable attention in both scientific research and clinical practice. Among these interventions, therapeutic taping is a strategy commonly employed to provide support to the ankle joint. The two most widely used methods are rigid tape (RT) and kinesiotape (KT).

RT has been shown to be effective in ankle stabilization, providing mechanical support to the joint and reducing the incidence of acute sprains [6]. However, potential side effects, such as restricted mobility in other regions of the body, have also been observed [7]. Specifically, increased knee valgus and greater tension in the patellar tendon have been reported, suggesting that ankle taping may induce global biomechanical changes in the lower limb, affecting the knee and hip [8,9]. Alterations in alignment and movement in the sagittal or frontal planes can modify load distribution patterns, predisposing runners to new injuries, as reported in previous studies [10].

KT, on the other hand, is an elastic tape that has gained popularity in the field of sports rehabilitation due to its potential benefits in proprioception and pain modulation [11,12]. Unlike RT, which restricts the joint’s range of motion, KT aims to provide support without significantly limiting mobility, potentially enhancing static joint stability and reducing the risk of recurrent injuries [13].

From a biomechanical perspective, several studies have evaluated the effects of both types of taping on joint mobility and sports performance, although the results are inconsistent. Halseth et al. [14] reported that the use of KT significantly reduced the ankle’s range of motion in healthy subjects, suggesting an improvement in joint stability. However, Briem et al. [15] found that RT was more effective than KT in limiting lateral ankle movement, and also observed significantly higher mean muscle activity when ankles were taped with RT. Nevertheless, neither method showed conclusive improvements in the prevention of recurrent sprains.

An important limitation in the existing literature is that most studies evaluate the effects of taping during isolated exercises, such as jumps or destabilization tests, which do not adequately reflect the prolonged and repetitive demands of activities like running [13,15]. Consequently, there is a need for studies that examine the long-term effects of taping during extended activities, such as running, to better understand its durability and efficacy [16]. Given that running is a repetitive activity, it is crucial to assess not only the immediate effects of taping but also its functionality after prolonged exertion. This includes evaluating the impact not only on the ankle joint but also on the entire lower limb to identify potential biomechanical compensations that could increase the risk of other injuries.

In biomechanical research studies, the combination of smartphones and the Kinovea software has proven to be a valid and reliable method for evaluating joint kinematics in different populations [17,18]. Kinovea is a free 2D motion analysis tool frequently used to measure kinematic parameters in activities such as running and vertical jumping [19].

Therefore, the present study aims to analyze the biomechanical effects of ankle taping using RT and KT, compared to no taping, in amateur runners over a 30 min treadmill run.

## 2. Materials and Methods

### 2.1. Study Design

A randomized, controlled, crossover clinical trial was conducted to analyze biomechanical changes in the lower limb as a result of ankle taping with RT and KT, compared to no taping, during treadmill running in amateur runners. The crossover design was chosen so that each participant could serve as their own control, thereby reducing inter-individual variability and increasing the precision of comparisons between the different conditions. Simple randomization was used to determine the order of the conditions for each subject, leaving a period of one week for washing.

Blinding was implemented for statistical analysis. The researcher who conducted the statistical analysis was blinded to the group assignments to eliminate any potential bias in the interpretation of the results.

The study followed the CONSORT guidelines and was carried out at the laboratory of the Faculty of Sport Sciences and Physiotherapy at the European University of Madrid between September and December 2023.

### 2.2. Participants

The study included 22 trained amateur runners (15 men and 7 women) with a mean age of 26.59 ± 9.25 years. The inclusion criteria were as follows: at least one year of experience in regular running practice, running a minimum of 20 km per week, and no lower limb injuries in the past 12 months. Participants were excluded if they had significant biomechanical abnormalities such as scoliosis or a leg length discrepancy, a history of lower limb surgeries, had previously regularly used ankle taping, or if they were pregnant. All participants provided written informed consent prior to the start of the study, which was approved by the Ethics Committee of the European University of Madrid (protocol no. XYZ-2023). Additionally, the study was registered with the Australian and New Zealand Clinical Trials Registry (ACTRN12624000099527).

Participant randomization was performed using the random function in Microsoft Excel (Microsoft Corporation, Redmond, WA, USA).

### 2.3. Interventions

Each participant underwent three treadmill running conditions following a randomized crossover design. Each intervention was performed by a certified physiotherapist with experience in taping techniques. It was ensured that the taping was correctly applied and consistently adhered in each session. 

#### 2.3.1. Running with KT Ankle Taping

**Figure 1:**
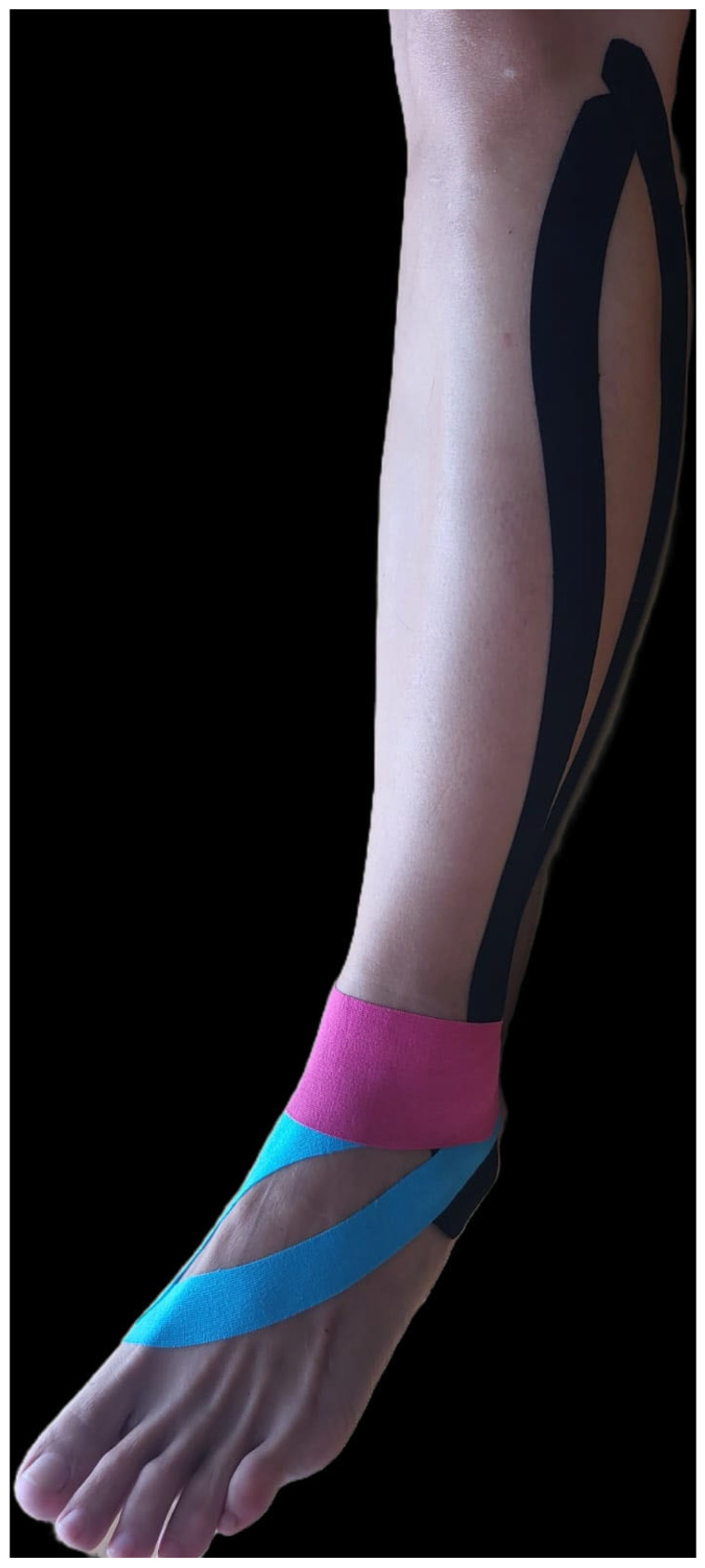
Kinesiotape ankle taping.

This technique involved the use of three segments of elastic KT: two “Y” strips and one “I” strip, each with a designated color for identification. The long “Y” strip (black) was applied from the lateral side of the calcaneus to the head of the fibula, covering the peroneus longus muscle. The short “Y” strip (light blue) was placed from the posterior side of the calcaneus to the base of the first metatarsophalangeal joint, ensuring comprehensive coverage of the targeted anatomical area. The “I” strip (pink) was applied longitudinally from the medial malleolus to the lateral malleolus, crossing the anterior aspect of the ankle (Figure 1) [20].

#### 2.3.2. Running with RT Ankle Taping

**Figure 2:**
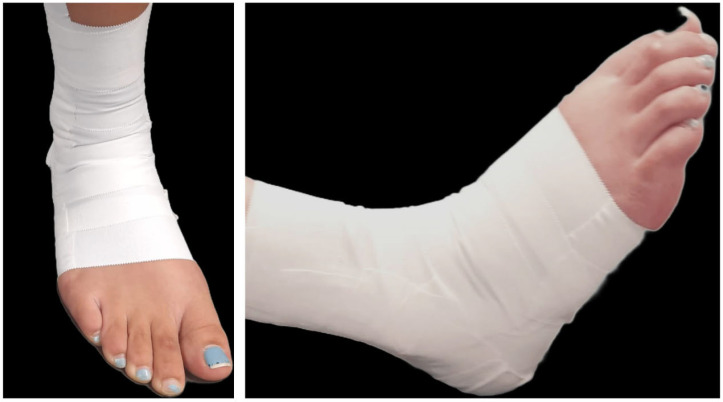
Rigid tape ankle taping.

The taping procedure was performed using conventional 38 mm adhesive tape. The application began by placing two anchor strips approximately 10 cm above the malleoli. Subsequently, two additional strips were applied from the medial edge of the anchor to the lateral side, with the foot positioned in a neutral stance. This was followed by creating “figure-six” configurations, starting from the medial anchor, wrapping under the foot, and returning to the same anchor. Finally, all ends and any loose areas were secured with additional tape to ensure a uniform and consistent application (Figure 2) [21].

#### 2.3.3. Control Group (CG)

The running session was performed without taping.

### 2.4. Adverse Events

In studies utilizing RT and KT taping as part of intervention protocols, it is crucial to consider the potential adverse effects that may arise both locally and systemically. Rigid tape, due to its non-elastic nature, can cause excessive compression that impairs blood and lymphatic circulation, thereby limiting oxygenation and the removal of metabolic byproducts in the underlying tissues. These effects may be exacerbated under conditions of muscular fatigue, compromising functional performance and contributing to the onset of pain or stiffness. Additionally, the partial immobilization imposed by rigid tape may alter natural movement patterns, increasing the risk of improper biomechanical compensation.

On the other hand, although KT serves as a more flexible and dynamic alternative, its use is not entirely free of side effects. In some cases, adverse skin reactions, such as irritation or contact dermatitis, have been reported, particularly in individuals with sensitive skin or during prolonged applications.

The potential adverse effects associated with the use of RT and KT described above were thoroughly explained to each participant during the informed consent process. It was ensured that each individual had a full understanding of the potential risks and benefits of the intervention. In this way, participant safety was safeguarded, and adherence to ethical principles in research was upheld.

### 2.5. Fatigue Protocol

Participants completed three running sessions, one for each condition, with a washout period of at least one week between sessions to minimize residual fatigue or adaptation effects [22].

**Figure 3:**
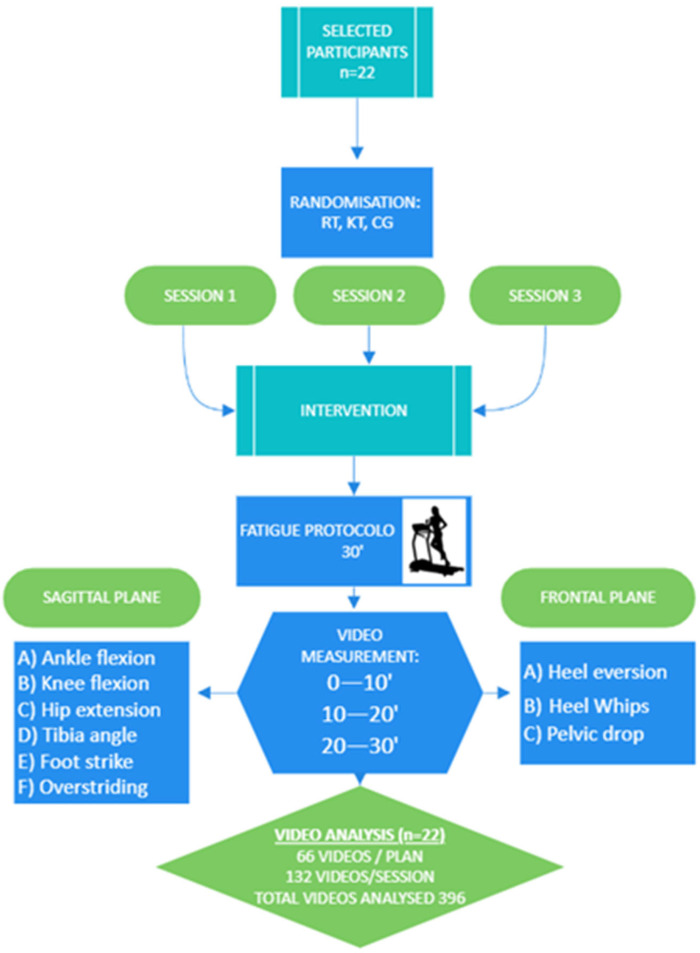
Flowchart of the study.

During each session, participants ran on a treadmill for 30 min at 85% of their maximum aerobic speed, which was previously determined through a maximal effort test [23]. The run was divided into three measurement intervals: 0–10, 10–20, and 20–30 min. Participants were recorded in the frontal and sagittal planes (Figure 3). Additionally, a 10 min warm-up consisting of mobility exercises and ballistic stretching was performed before the start of each running session. 

The maximal effort test consisted of running at maximum effort for 5 min on a 400 m track, measuring the total distance covered, and calculating the maximum aerobic speed (MAS) by dividing the distance by the time.

### 2.6. Data Collection

The Kinovea biomechanical analysis software (GPLv2 license) was used to perform the kinematic analysis of the runners’ movements in the frontal and sagittal planes. Reflective markers were placed on key anatomical points of the lower limb following a standard kinematic analysis protocol: in the sagittal plane—hip (greater trochanter), knee (lateral epicondyle), ankle (lateral malleolus), and foot; and in the frontal plane—posterior superior iliac spines (PSIS), midpoint of the popliteal fossa, and midpoint of the calcaneal dome. Recordings were captured using two high-speed (240 fps) and high-resolution (1080 × 1920 px) cameras positioned at the frontal and lateral views of the treadmill, at a distance of 3 m. The data were subsequently analyzed to assess joint angles in the frontal and sagittal planes [24].

**Figure 4:**
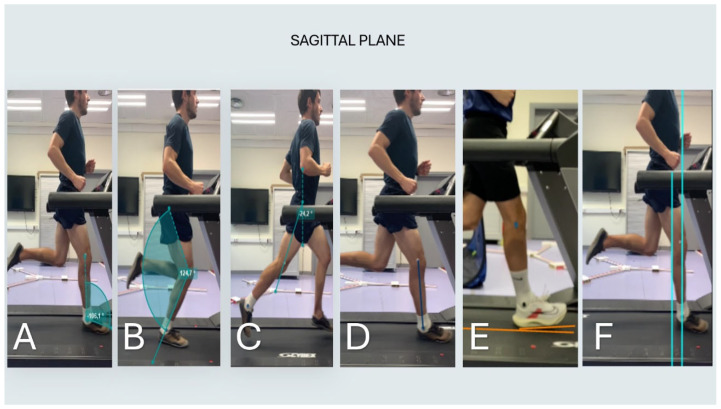
(**A**) Ankle flexion at initial contact, (**B**) knee flexion during stance, (**C**) hip extension during late phase, (**D**) tibial angle during the stance phase, (**E**) foot strike pattern, (**F**) overstriding.

The following biomechanical variables were measured in the sagittal plane: ankle dorsiflexion angle at initial contact, maximum knee flexion angle during the stance phase, maximum hip extension angle during the late phase, tibial angle during the stance phase, and the foot strike pattern [24] (Figure 4). Cadence was also assessed (steps/min).

- Ankle dorsiflexion angle at initial contact: This angle was measured by drawing a line from the midpoint of the lateral malleolus to the head of the fifth metatarsal, and another line from the midpoint of the lateral malleolus to the lateral condyle of the tibia. The angle formed between these two lines at the moment of initial ground contact indicates the degree of ankle dorsiflexion.
- Maximum knee flexion angle during the stance phase: The knee flexion angle was assessed by drawing a line from the greater trochanter to the lateral epicondyle of the femur and another line from the lateral epicondyle to the lateral malleolus. The maximum angle formed between these two lines during the stance phase represents the peak knee flexion.
- Maximum hip extension angle during the late phase: The hip extension angle was measured by drawing one line vertically and another line from the greater trochanter to the lateral epicondyle of the femur. The maximum angle formed between these two lines, as the leg extends behind the body during the late phase of movement, indicates peak hip extension.
- Tibial angle during the stance phase: This angle was measured by drawing a line from the midpoint of the lateral malleolus to the lateral condyle of the tibia. The angle between this line and the vertical axis represents the tibial angle, which provides information on tibial inclination during the stance phase. The tibial angle was then classified as either extension or neutral.
- Foot strike pattern: Foot strike pattern was determined by evaluating the point of initial contact between the foot and the ground, using the relative position of the calcaneus and metatarsal heads. The initial contact was classified as heel strike, midfoot strike, or forefoot strike depending on which region of the foot made first contact with the ground.
- Overstriding: Overstriding in runners was assessed during the loading response phase by drawing a vertical line from the lateral malleolus to evaluate its alignment with the pelvis. Two categories were defined: overstriding, where the vertical line appeared anterior to the pelvis, and no overstriding, where the vertical line fell within the pelvis.

**Figure 5:**
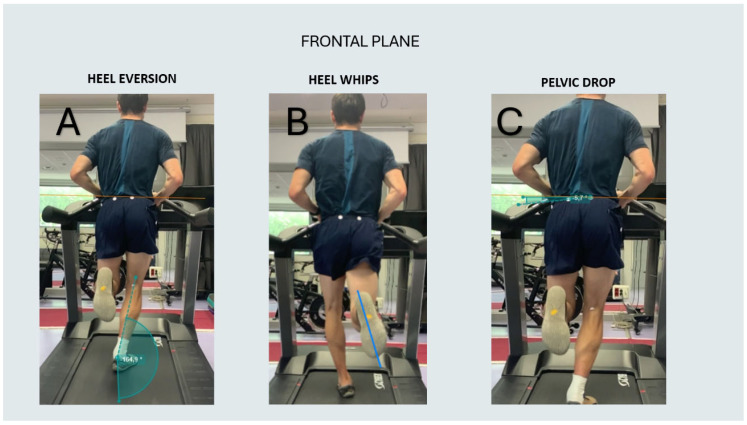
(**A**) Heel eversion in the mid-stance phase, (**B**) heel whips during the swing phase, (**C**) pelvic drop during the swing phase.

In the frontal plane, the following biomechanical variables were measured: heel eversion angles during the mid-stance phase, heel whips during swing phase, and pelvic tilt [24] (Figure 5).

- Heel eversion was measured by identifying the mid-stance phase by placing markers at the top and bottom of the heel counter of the shoe. One line was drawn from the midpoint of the popliteal fossa to the top of the heel counter, and another line was drawn from the top to the bottom of the heel counter. The angle formed between these two lines represents the degree of heel eversion relative to the leg.
- Heel whips were assessed by tracking the rotation of the heel relative to the forefoot during the swing phase of the gait cycle. To measure this, a line was drawn from the midpoint of the calcaneus to the midpoint of the forefoot, and its displacement in the medial or lateral direction was recorded throughout the swing phase. Based on this analysis, runners were categorized into medial or lateral heel whips. Three groups were categorized based on medial deviation: less than 5 degrees, between 5 and 10 degrees, and greater than 10 degrees, as well as a lateral category of less than 5 degrees.
- Pelvic tilt was evaluated by marking the anterosuperior iliac spines (ASIS) on both sides of the pelvis and drawing a horizontal line between them. The angle between this reference line and the horizontal plane represents the degree of pelvic tilt.

### 2.7. Statistical Analysis

Statistical analyses were performed using SPSS version 29 for Windows. The normality of the data was assessed using the Shapiro–Wilk test (*n* = 22 participants per group). Continuous variables are presented as mean and standard deviation (SD), while categorical variables are reported as frequencies and percentages. A repeated measures ANOVA (3 × 3) was conducted to analyze differences between the three groups (CG, RT and KT) and across the three measurement intervals (0–10, 10–20, and 20–30 min). Assumptions of sphericity were tested using Mauchly’s test, and homoscedasticity and heterogeneity were evaluated through Levene’s test and residual plots. No post hoc tests were performed, as none of the models showed significant effects for any variable. Effect sizes were estimated using partial eta squared (η^2^p), with values interpreted as small (0.01), medium (0.06), and large (0.14). Chi-square tests were used to assess group differences in the distribution of categorical variables. All graphical representations were created using GraphPad Prism. Statistical significance was set at *p* < 0.05.

## 3. Results

Descriptive statistics for the participants (*n* = 22; 17 male and 5 female) are presented as mean ±SD. The mean age of the participants was 26.59 ± 9.25 years. The average weight was 66.14 ± 8.17 kg, and the mean height was 1.74 ± 0.06 m. The average BMI was 21.83 ± 1.67 kg/m^2^. In the 5 min test, participants covered an average distance of 1172 ± 167 m. The mean aerobic maximal speed was 3.90 ± 0.55 m/s. During the velocity fatigue protocol, the average speed reached was 11.9 ± 1.69 km/h. The mean weekly training volume was 27.3 ± 10.6 km. Non adverse events were observed during the running protocol with or without taping procedures.

### 3.1. Sagittal Plane

**Figure 6:**
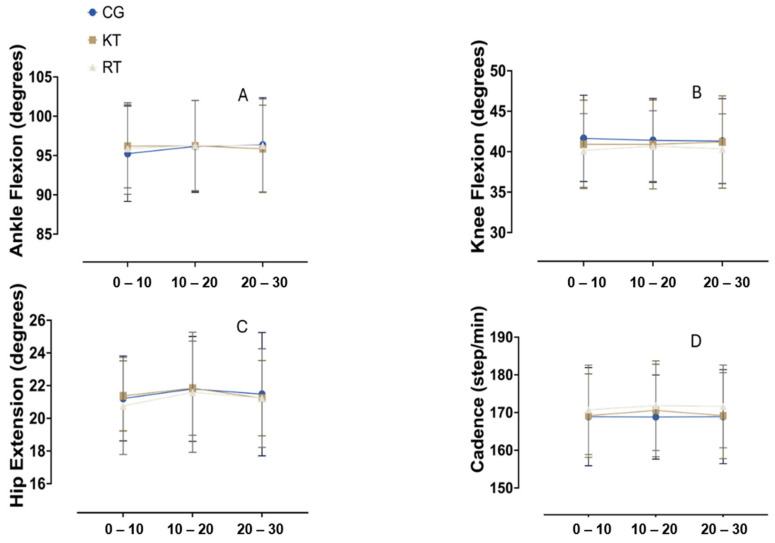
Sagittal plane kinematic variables during running for different interventions. (**A**) Ankle flexion (degrees), (**B**) knee flexion (degrees), (**C**) hip extension (degrees), and (**D**) cadence (steps/min) for all groups across the time intervals of 0–10, 10–20, and 20–30 min. CG, control group; KT, kinesiotape group; RT, rigid tape group.

The repeated measures ANOVA results did not show a significant main effect of time on ankle flexion across the three intervals (0–10, 10–20, and 20–30 min), F (1, 63) = 1.387, *p* = 0.243 and partial eta squared (η^2^) = 0.022. Additionally, no significant differences were found between groups (CG, RT and KT), F (2, 63) = 0.010, *p* = 0.990, η^2^ = 0.000. The interaction between time and group was also non-significant, F (2, 126) = 1.610, *p* = 0.208, η^2^ = 0.049 (Figure 6A). In terms of knee flexion, there was no significant main effect of time, F (1, 63) = 0.029, *p* = 0.866, η^2^ = 0.000, or group, F (2, 63) = 0.251, *p* = 0.779, η^2^ = 0.008. The time by group interaction was non-significant, F (2, 126) = 0.505, *p* = 0.606, η^2^ = 0.016 (Figure 6B). For hip extension, no significant effect of time was observed, F (1, 63) = 0.537, *p* = 0.466, η^2^ = 0.008, indicating no changes over the measurement intervals. Similarly, no significant group differences were found, F (2, 63) = 0.085, *p* = 0.919, η^2^ = 0.003, nor was there a significant time by group interaction, F (2, 126) = 0.455, *p* = 0.637, η^2^ = 0.014 (Figure 6C). Cadence analysis indicated no significant main effect of time, F (1, 63) = 0.330, *p* = 0.568, η^2^ = 0.005, or group, F (2, 63) = 0.277, *p* = 0.759, η^2^ = 0.009. The time by group interaction effect was also non-significant, F (2, 126) = 0.330, *p* = 0.720, η^2^ = 0.010 (Figure 6D). The Supplementary File shows the means and standard deviation of all groups and the time of measurement.

The chi-square analysis for foot strike pattern and tibia angle did not show significant differences between the CG, RT and KT groups across the different time intervals (0–10, 10–20, and 20–30 min) (Table 1). Similarly, for overstriding, all participants in each group consistently exhibited overstriding (*n* = 22) across all intervals, with no variation, resulting in no calculated chi-square values due to the constant nature of the variable.

### 3.2. Frontal Plane

**Figure 7:**
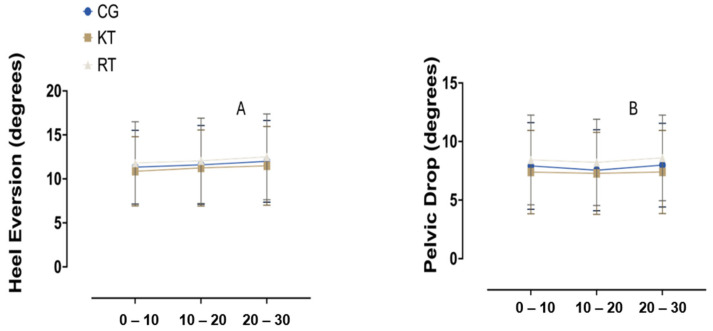
Heel eversion and pelvic drop across time intervals for different interventions. (**A**) Heel eversion (degrees) at 0–10, 10–20 and 20–30 min intervals, (**B**) Pelvic drop (degrees) across the same time intervals and groups. CG, control group; RT, rigid tape group; KT, kinesiotape group.

Regarding the frontal plane measurements, the repeated measures ANOVA results show a significant main effect of time on heel eversion across the three intervals, F (2, 63) = 6.706, *p* = 0.012, partial eta squared (η^2^) = 0.096. Additionally, no significant differences were found between groups (CG, RT and KT), F (2, 63) = 0.608, *p* = 0.548, η^2^ = 0.019. The interaction between time and group was also non-significant, F (4, 126) = 0.298, *p* = 0.743, η^2^ = 0.009 (Figure 7A). In terms of pelvic drop, there was no significant main effect of time, F (2, 63) = 0.038, *p* = 0.847, η^2^ = 0.001, or group, F (2, 63) = 0.048, *p* = 0.945, η^2^ = 0.002. The time by group interaction was non-significant, F (4, 126) = 0.809, *p* = 0.450, η^2^ = 0.025 (Figure 7B). The Supplementary File shows the means and standard deviation of all groups and the time of measurement.

The chi-square analysis for the heel whip distribution did not show significant differences between the CG, RT and KT groups across the different time intervals (*p* > 0.05) (Table 1).

## 4. Discussion

This novel research analyzed the effects of RT and KT on running kinematics in well-trained runners following a treadmill-based fatigue protocol. This protocol simulated real-world fatigue conditions during running due to its duration and intensity, comparable to those experienced in competitive events. The hypothesis was that RT and KT would significantly influence biomechanical variables in both the sagittal (e.g., ankle flexion, knee flexion, hip extension, tibia angle, foot strike pattern, and cadence) and frontal plane (e.g., heel eversion, heel whips, and pelvic drop). To achieve this, a previously validated 2D motion analysis system was employed [25,26]. Additionally, the aim was to analyze whether the observed biomechanical differences between groups would remain consistent at different time intervals (0–10, 10–20, and 20–30 min), as recent studies have suggested differential biomechanical responses to prolonged tape use [27].

### 4.1. Sagittal Plane

Ankle flexion did not differ significantly between groups, contrasting with findings from other studies on individuals with CAI [28] and studies that restricted ankle range of motion using KT or RT [13,14,15] examining isolated tasks, such as jumping or balance tests, which may not fully capture the prolonged and repetitive demands inherent to activities like running. Nevertheless, no significant differences were observed between pre- and post-fatigue analyses in the present study, suggesting that ankle flexion was unaffected by the fatigue protocol.

Ankle flexion is a key determinant of foot strike pattern, and consistent with this, the foot strike pattern remained unchanged across the different time intervals and between groups. The same was observed for knee flexion during stance, hip extension during late stance, tibia angle during the stance phase, and foot strike pattern. Based on previous studies, we hypothesized that both RT and KT could restrict ankle range of motion and alter lower limb muscle activation [14,15] potentially leading to changes in running biomechanics. For instance, limiting ankle dorsiflexion could influence foot strike pattern, and tibia angle [28,29].

The lack of significant differences between groups in the ankle flexion and foot strike pattern may also explain the absence of changes in knee flexion. A recent meta-analysis highlighted the strong association between the foot strike pattern and knee flexion angle during running. Specifically, forefoot strikers tend to have a plantar-flexed ankle and a more flexed knee at initial contact, compared to rearfoot strikers, who exhibit a dorsiflexed ankle and a more extended knee. Midfoot strikers demonstrated greater ankle dorsiflexion and decreased knee flexion compared to rearfoot strikers [30].

Similarly, the absence of significant differences in the knee flexion during stance may explain why no differences were observed in hip extension during late stance, as these two variables are closely linked [29,31]. This relationship also extends to overstriding, which is associated with hip extension; the lack of group differences in hip extension corresponded with no changes in overstriding [32]. Finally, as overstriding, hip extension, and running speed (fixed by the protocol) were unaffected, cadence remained unchanged [29]. Thus, the null hypothesis was supported for sagittal plane variables, with no significant biomechanical differences between groups or across time. In contrast, a recent meta-analysis reported that ankle taping influences sagittal plane biomechanics in runners; however, this population had CAI, and the observed modifications were characterized by low to very low certainty [33].

### 4.2. Frontal Plane

Heel eversion showed no significant differences between groups across time intervals during the fatigue protocol, in contrast to other studies performed with subjects with CAI or chronic ankle sprains [34,35]. Nevertheless, our results are consistent with a recent meta-analysis on frontal plane ankle kinematics [33]. Across all groups, heel eversion significantly improved over time, indicating that this variable was influenced by the fatigue protocol. Similar findings were reported in novice runners following a treadmill-based fatigue protocol [36].

In the same line, there were no differences in the heel whips analysis between groups across the different time intervals (0–10 min, 10–20 min, 20–30 min). No significant differences were observed in the distribution of heel deviation types (medial or lateral). The data revealed a consistent trend within each group, suggesting that the application of taping did not influence running biomechanics with respect to this parameter. Throughout the three intervals, the distribution of heel whip patterns remained similar across the groups. In the initial 0–10 min interval, both the CG and KT groups demonstrated a higher prevalence of medial deviations greater than 10 degrees (59.1% and 63.6%, respectively), while the RT group exhibited an even higher prevalence in this category (77.3%). This trend persisted across the 10–20 and 20–30 min intervals, with no significant changes observed in any of the categories (medial < 5°, medial 5–10°, medial > 10°, and lateral < 5°). These findings suggest that neither fatigue nor the type of taping used had a significant impact on the magnitude of heel whips in well-trained runners during continuous running.

Regarding pelvic drop, no significant differences were found in the pre-post analysis, indicating that it was unaffected by the fatigue protocol. Pelvic drop during running is closely related to hip abductor and hip extensor strength. It is plausible that the well-trained runners in our study had sufficient hip strength to prevent alterations in pelvic drop despite fatigue [37]. Additionally, no significant differences were observed between groups. This finding aligns with recent meta-analyses that concluded that ankle taping does not significantly affect frontal plane running biomechanics [33].

## 5. Clinical Implications, Limitations and Future Lines of Research 

While this study offers novel insights, several limitations must be acknowledged. First, this study was a secondary analysis from broader research, so there was not a specific sample size calculation, and perhaps the sample size was not large enough. Second, the structural analysis of the foot was not considered, but this may be relevant to future research. The absence of significant biomechanical differences between groups and across time suggests that the treadmill protocol may not have induced enough muscular fatigue in these well-trained runners to alter running biomechanics. A more prolonged or intense protocol, or one conducted on uneven, overground surfaces, might be necessary, as running biomechanics can differ between treadmill and overground conditions, particularly in the sagittal plane [38]. Furthermore, the lack of significant differences in running biomechanics between taping conditions does not preclude the possibility that RT and KT could affect other variables, such as muscle activation [39].

In this sense, our findings contrast with previous studies that have reported biomechanical changes associated with the use of taping, particularly during tasks with higher biomechanical demands such as jumping and landing. For example, the study by Romero-Morales et al. (2024) demonstrated that prophylactic ankle RT influenced ankle and knee biomechanics during landing tasks, suggesting that RT may be more effective in altering lower limb kinematics in movements that involve greater mechanical load and increased neuromuscular control, such as jump landings, compared to continuous running [21]. Similarly, Cheraghi et al. (2022) observed that the use of KT in collegiate athletes with CAI led to changes in ankle kinematics during the landing phase of a single leg drop landing. Their results indicated that taping could modify plantarflexion, dorsiflexion, ankle flexion, and tibial angle [40]. These findings suggest that taping may influence force distribution and neuromuscular control of the lower limb during high-demand movements. One possible explanation for these differences is that our participants were healthy, well-trained runners with no history of chronic instability or biomechanical alterations, which may account for the lack of changes in heel kinematics during running.

Thus, the findings of this study do not rule out the potential utility of RT or KT in providing ankle stability or serving as a preventive strategy within a holistic injury prevention program. Future research should explore the effects of more demanding fatigue protocols or overground conditions, with a focus on variables like electromyographic activity and ankle stability. Finally, as this study involved well-trained runners, it would be interesting to compare these results with novice or elite runners.

## Figures and Tables

**Table 1:** Distribution of heel whips, tibia angulation, and foot strike patterns across time intervals and groups.

Interval (Minutes)	Group	Heel Whips (Frontal Plane)				Chi-Square (*p*-Value)	Tibia Angle (Sagital Plane)		Chi-Square (*p*-Value)	Foot Strike (Sagital Plane)			Chi-Square (*p*-Value)
		Medial (<5)	Medial 5–10	Medial >10	Lateral <5		Extension	Neutral		Heel strike	Midfoot	Forefoot	
0–10	CG	2 (9.1%)	6 (27.3%)	13 (59.1%)	1 (4.5%)	χ^2^ = 2.591 (*p* = 0.858)	19 (86.4%)	3 (13.6%)	χ^2^ = 0.236 (*p* = 0.889)	17 (77.3%)	3 (13.6%)	2 (9.1%)	χ^2^ = 0.668 (*p* = 0.953)
	KT	2 (9.1%)	5 (22.7%)	14 (63.6%)	1 (4.5%)		19 (86.4%)	3 (13.6%)		18 (81.8%)	2 (9.1%)	2 (9.1%)	
	RT	2 (9.1%)	2 (9.1%)	17 (77.3%)	1 (4.5%)		18 (81.8%)	4 (18.2%)		18 (81.8%)	3 (13.6%)	1 (4.5%)	
10–20	CG	2 (9.1%)	5 (22.7%)	14 (63.6%)	1 (4.5%)	χ^2^ = 1.415 (*p* = 0.965)	19 (86.4%)	3 (13.6%)	χ^2^ = 0.236 (*p* = 0.889)	17 (77.3%)	4 (18.2%)	1 (4.5%)	χ^2^ = 1.220 (*p* = 0.875)
	KT	2 (9.1%)	5 (22.7%)	14 (63.6%)	1 (4.5%)		19 (86.4%)	3 (13.6%)		18 (81.8%)	3 (13.6%)	1 (4.5%)	
	RT	1 (4.5%)	3 (13.6%)	17 (77.3%)	1 (4.5%)		18 (81.8%)	4 (18.2%)		18 (81.8%)	4 (18.2%)	0 (0%)	
20–30	CG	1 (4.5%)	6 (27.3%)	14 (63.6%)	1 (4.5%)		19 (86.4%)	3 (13.6%)	χ^2^ = 0.236 (*p* = 0.889)	18 (81.8%)	3 (13.6%)	1 (4.5%)	χ^2^ = 0.321 (*p* = 0.988)
	KT	2 (9.1%)	5 (22.7%)	14 (63.6%)	1 (4.5%)	χ^2^ = 1.900 (*p* = 0.929)	19 (86.4%)	3 (13.6%)		19 (86.4%)	2 (9.1%)	1 (4.5%)	
	RT	1 (4.5%)	3 (13.6%)	17 (77.3%)	1 (4.5%)		18 (81.8%)	4 (18.2%)		19 (86.4%)	2 (9.1%)	1 (4.5%)	

Abbreviatures: CG, control group; KT, kinesiotape group; RT, rigid tape group. Results are presented as frequency (percentage).

## Data Availability

The data that support the findings of this study are available on request from the corresponding author, Javier López-Ruiz, javier.lopez3@universidadeuropea.es.

## Supplementary Materials

The following supporting information can be downloaded at: https://www.mdpi.com/article/10.3390/jcm13226740/s1, Table S1: Summary of Repeated Measures ANOVA Results for Sagittal Plane Kinematic Variables During Running; Table S2: Summary of Repeated Measures ANOVA Results for Sagittal Frontal Kinematic Variables During Running.

## References

[1] Teixeira R.N., Lunardi A., da Silva R.A., Lopes A.D., Carvalho C.R. (2016). Prevalence of musculoskeletal pain in marathon runners who compete at the elite level. Int. J. Sports Phys. Ther..

[2] Van Gent R.N., Siem D., Van Middelkoop M., Van Os A.G., Bierma-Zeinstra S.M.A., Koes B.W. (2007). Incidence and determinants of lower extremity running injuries in long distance runners: A systematic review. Br. J. Sports Med..

[3] Fong D.T.P., Hong Y., Chan L.K., Yung P.S.H., Chan K.M. (2007). A systematic review on ankle injury and ankle sprain in sports. Sports Med..

[4] Yin Y., Yu Z., Wang J., Sun J. (2022). Effectiveness of the Rehabilitation Training Combined with Maitland Mobilization for the Treatment of Chronic Ankle Instability: A Randomized Controlled Trial. Int. J. Environ. Res. Public Health.

[5] Gribble P.A., Bleakley C.M., Caulfield B.M., Docherty C.L., Fourchet F., Fong D.T.P., Hertel J., Hiller C.E., Kaminski T.W., McKeon P.O. (2016). 2016 consensus statement of the International Ankle Consortium: Prevalence, impact and long-term consequences of lateral ankle sprains. Br. J. Sports Med..

[6] Ghai S., Ghai I., Narciss S. (2024). Influence of taping on joint proprioception: A systematic review with between and within group meta-analysis. BMC Musculoskelet. Disord..

[7] Williams S.A., Ng L., Stephens N., Klem N., Wild C. (2018). Effect of prophylactic ankle taping on ankle and knee biomechanics during basketball-specific tasks in females. Phys. Ther. Sport.

[8] Rao Y., Yang N., Gao T., Zhang S., Shi H., Lu Y., Ren S., Huang H. (2023). Effects of peak ankle dorsiflexion angle on lower extremity biomechanics and pelvic motion during walking and jogging. Front. Neurol..

[9] Backman L.J., Danielson P. (2011). Low Range of Ankle Dorsiflexion Predisposes for Patellar Tendinopathy in Junior Elite Basketball Players: A 1-Year Prospective Study. Am. J. Sports Med..

[10] Schiltz M., dric Lehance C., Maquet D., Bury T., Crielaard J.M., Croisier J.L. (2009). Explosive Strength Imbalances in Professional Basketball Players. J. Ath. Train.

[11] Tudini F., Jordon M., Levine D., Healy M., Cathey S., Chui K. (2024). Evaluating the effects of two different kinesiology taping techniques on shoulder range of motion and proprioception in patients with hypermobile Ehlers–Danlos syndrome: A randomized controlled trial. Front. Rehabil. Sci..

[12] Heddon S., Saulnier N., Mercado J., Shalmiyev M., Berteau J.P. (2021). Systematic review shows no strong evidence regarding the use of elastic taping for pain improvement in patients with primary knee osteoarthritis. Medicine.

[13] Watcharakhueankhan P., Chapman G.J., Sinsurin K., Jaysrichai T., Richards J. (2022). The immediate effects of Kinesio Taping on running biomechanics, muscle activity, and perceived changes in comfort, stability and running performance in healthy runners, and the implications to the management of Iliotibial band syndrome. Gait Posture.

[14] Halseth T., McChesney J.W., Debeliso M., Vaughn R., Lien J. (2004). The effects of kinesio™ taping on proprioception at the ankle. J. Sci. Med. Sport.

[15] Briem K., Eythörsdöttir H., Magnúsdóttir R.G., Pálmarsson R., Rúnarsdöttir T., Sveinsson T. (2011). Effects of Kinesio Tape compared with nonelastic sports tape and the untaped ankle during a sudden inversion perturbation in male athletes. J. Orthop. Sports Phys. Ther..

[16] Cordova M.L., Ingersoll C.D., Palmieri R.M. (2002). Efficacy of Prophylactic Ankle Support: An Experimental Perspective. J. Athl. Train.

[17] Fernández-González P., Koutsou A., Cuesta-Gómez A., Carratalá-Tejada M., Miangolarra-Page J.C., Molina-Rueda F. (2020). Reliability of kinovea® software and agreement with a three-dimensional motion system for gait analysis in healthy subjects. Sensors.

[18] Puig-Diví A., Escalona-Marfil C., Padullés-Riu J.M., Busquets A., Padullés-Chando X., Marcos-Ruiz D. (2019). Validity and reliability of the Kinovea program in obtaining angles and distances using coordinates in 4 perspectives. PLoS ONE.

[19] Damsted C., Nielsen R.O., Larsen L.H. (2015). Reliability of video-based quantification of the knee- and hip angle at foot strike during running. Int. J. Sports Phys. Ther..

[20] Sarvestan J., Ataabadi P.A., Svoboda Z., Kovačikova Z., Needle A.R. (2020). The effect of ankle KinesioTM taping on ankle joint biomechanics during unilateral balance status among collegiate athletes with chronic ankle sprain. Phys. Ther. Sport.

[21] Romero-Morales C., Matilde-Cruz A., García-Arrabe M., Higes-Núñez F., Lópes A.D., Saiz S.J., Pareja-Galeano H., López-López D. (2024). Assessing the effect of prophylactic ankle taping on ankle and knee biomechanics during landing tasks in healthy individuals: A cross-sectional observational study. Sao Paulo Med. J..

[22] Slevin Z.M., Arnold G.P., Wang W., Abboud R.J. (2020). Immediate effect of kinesiology tape on ankle stability. BMJ Open Sport Exerc. Med..

[23] García-Pérez J.A., Pérez-Soriano P., Llana Belloch S., Lucas-Cuevas Á.G., Sánchez-Zuriaga D. (2014). Effects of treadmill running and fatigue on impact acceleration in distance running. Sports Biomech..

[24] Souza R.B. (2016). An Evidence-Based Videotaped Running Biomechanics Analysis. Phys. Med. Rehabil. Clin. N. Am..

[25] Pipkin A., Kotecki K., Hetzel S., Heiderscheit B. (2016). Reliability of a qualitative video analysis for running. J. Orthop. Sports Phys. Ther..

[26] Atkins L.T., James C.R., Sizer P.S., Jonely H., Brismée J.M. (2014). Reliability and concurrent criterion validity of a novel technique for analyzing hip kinematics during running. Physiother. Theory Pract..

[27] Utku B., Bähr G., Knoke H., Mai P., Paganini F., Hipper M., Braun L., Denis Y., Helwig J., Willwacher S. (2024). The effect of fresh and used ankle taping on lower limb biomechanics in sports specific movements. J. Sci. Med. Sport.

[28] Chinn L., Dicharry J., Hart J.M., Saliba S., Wilder R., Hertel J. (2014). Gait kinematics after taping in participants with chronic ankle instability. J. Athl. Train.

[29] Kwon Y., Shin G. (2022). Foot kinematics and leg muscle activation patterns are altered in those with limited ankle dorsiflexion range of motion during incline walking. Gait Posture.

[30] Almeida M.O., Davis I.S., Lopes A.D. (2015). Biomechanical Differences of Foot-Strike Patterns During Running: A Systematic Review With Meta-analysis. J. Orthop. Sports Phys. Ther..

[31] Moffit T.J., Montgomery M.M., Lockie R.G., Pamukoff D.N. (2020). Association Between Knee- and Hip-Extensor Strength and Running-Related Injury Biomechanics in Collegiate Distance Runners. J. Athl. Train.

[32] Baker L.M., Yawar A., Lieberman D.E., Walsh C.J. (2024). Predicting overstriding with wearable IMUs during treadmill and overground running. Sci. Rep..

[33] Rowe P.L., Bryant A.L., Egerton T., Paterson K.L. (2023). External Ankle Support and Ankle Biomechanics in Chronic Ankle Instability: Systematic Review and Meta-Analysis. J. Athl. Train.

[34] Gregory C., Koldenhoven R.M., Higgins M., Hertel J. (2019). External ankle supports alter running biomechanics: A field-based study using wearable sensors. Physiol. Meas..

[35] Deschamps K., Dingenen B., Pans F., Van Bavel I., Matricali G.A., Staes F. (2016). Effect of taping on foot kinematics in persons with chronic ankle instability. J. Sci. Med. Sport.

[36] Koblbauer I.F., van Schooten K.S., Verhagen E.A., van Dieën J.H. (2014). Kinematic changes during running-induced fatigue and relations with core endurance in novice runners. J. Sci. Med. Sport.

[37] Tsatalas T., Giakas G., Spyropoulos G., Sideris V., Lazaridis S., Kotzamanidis C., Koutedakis Y. (2013). The effects of eccentric exercise-induced muscle damage on running kinematics at different speeds. J. Sports Sci..

[38] Van Hooren B., Fuller J.T., Buckley J.D., Miller J.R., Sewell K., Rao G., Barton C., Bishop C., Willy R.W. (2020). Is Motorized Treadmill Running Biomechanically Comparable to Overground Running? A Systematic Review and Meta-Analysis of Cross-Over Studies. Sports Med..

[39] Biz C., Nicoletti P., Tomasin M., Bragazzi N.L., Di Rubbo G., Ruggieri P. (2022). Is Kinesio Taping Effective for Sport Performance and Ankle Function of Athletes with Chronic Ankle Instability (CAI)? A Systematic Review and Meta-Analysis. Medicina.

[40] Cheraghi M., Boozari S., Svoboda Z., Kovačikova Z., Needle A.R., Sarvestan J. (2022). Effects of ankle KinesioTM taping on jump biomechanics in collegiate athletes with chronic ankle instability. Sport Sci. Health.

