# Supplementary material for: Ankle Taping Does Not Affect Running Kinematics During a Treadmill Protocol in Well-Trained Runners: A Secondary Analysis from a Randomized Cross-Over Controlled Trial
Source: J Clin Med. 2024 Nov 8;13(22):6740. doi: 10.3390/jcm13226740 (PMC11594344; doi:10.3390/jcm13226740)
Supplement: Supplementary file 1 [file jcm-13-06740-s001.zip › jcm-3270595-supplementary.pdf]

Table S1. Summary of Repeated Measures ANOVA Results for Sagittal Plane Kinematic Variables During Running.

Variable	Group	Interval (minutes)			Time F; P (Eta^2)	Group F; P (Eta^2)	Group*Time F; P (Eta^2)
		0-10	10-20	20-30			
ANKLE FLEXION	CG	95.24 ± 6.07	96.17 ± 5.85	96.36 ± 5.99	F = 1.387; p = 0.243 (0.022)	F = 0.010; p = 0.990 (0.000)	F = 1.610; p = 0.208 (0.049)
	KT	96.19 ± 5.31	96.23 ± 5.79	95.86 ± 5.56			
	RT	95.90 ± 5.82	96.27 ± 5.74	96.28 ± 5.91			
KNEE FLEXION	CG	41.65 ± 5.33	41.39 ± 5.19	41.31 ± 5.24	F = 0.029; p = 0.866 (0.000)	F = 0.251; p = 0.779 (0.008)	F = 0.505; p = 0.606 (0.016)
	KT	40.92 ± 5.46	40.90 ± 5.50	41.19 ± 5.70			
	RT	40.15 ± 4.56	40.69 ± 4.38	40.34 ± 4.33			
HIP EXTENSION	CG	21.22 ± 2.60	21.80 ± 3.21	21.48 ± 3.78	F = 0.537; p = 0.466 (0.008)	F = 0.085; p = 0.919 (0.003)	F = 0.455; p = 0.637 (0.014)
	KT	21.37 ± 2.14	21.84 ± 2.88	21.23 ± 2.30			
	RT	20.75 ± 2.97	21.59 ± 3.67	21.24 ± 3.02			
CADENCE	CG	168.91 ± 13.00	168.82 ± 11.16	168.91 ± 12.46	F = 0.330; p = 0.568 (0.005)	F = 0.277; p = 0.759 (0.009)	F = 0.330; p = 0.720 (0.010)
	KT	169.18 ± 11.05	170.55 ± 12.25	169.18 ± 11.39			
	RT	170.73 ± 11.87	171.82 ± 11.87	171.64 ± 10.97			

Abbreviatures: CG, control group; KT, kinesiotape group; RT, rigid tape group.

Table S2. Summary of Repeated Measures ANOVA Results for Sagittal Frontal Kinematic Variables During Running.

Variable	Group	Interval (minutes)			Time<br>F; P (Eta^2)	Group<br>F; P (Eta^2)	Group*Time<br>F; P (Eta^2)
		0-10	10-20	20-30			
HEEL EVERSION	CG	11.33 ± 4.18	11.59 ± 4.47	11.99 ± 4.64	F = 6.706; p = 0.012 (0.096)	F = 0.608; p = 0.548 (0.019)	F = 0.298; p = 0.743 (0.009)
	KT	10.86 ± 3.93	11.24 ± 4.32	11.48 ± 4.47			
	RT	11.80 ± 4.69	12.05 ± 4.85	12.50 ± 4.88			
PELVIC DROP	CG	7.91 ± 3.70	7.55 ± 3.46	7.98 ± 3.57	F = 0.038; p = 0.847 (0.001)	F = 0.048; p = 0.945 (0.002)	F = 0.809; p = 0.450 (0.025)
	KT	7.39 ± 3.56	7.28 ± 3.50	7.40 ± 3.55			
	RT	8.43 ± 3.83	8.22 ± 3.68	8.60 ± 3.66			

Abbreviatures: CG, control group; KT, kinesiotape group; RT, rigid tape group.
